# Contemporary Diabetes Technology and the Patient Experience

**DOI:** 10.1001/jamanetworkopen.2026.24260

**Published:** 2026-07-21

**Authors:** Estelle M. Everett, Erin Shaw, Bryan Escobar Barrios, Tannaz Moin, Lauren E. Wisk

**Affiliations:** 1 Division of Endocrinology, Diabetes, & Metabolism, Department of Medicine, David Geffen School of Medicine, University of California, Los Angeles; 2 Division of General Internal Medicine & Health Services Research, Department of Medicine, David Geffen School of Medicine, University of California, Los Angeles; 3 Veterans Affairs Greater Los Angeles Healthcare System, Los Angeles, California; 4 Department of Health Policy and Management, Fielding School of Public Health, University of California, Los Angeles

## Abstract

**Importance:**

Use of insulin pumps, continuous glucose monitors (CGMs), and automated insulin delivery (AID) systems has increased substantially in type 1 diabetes (T1D) care. Although these technologies improve glycemic outcomes, their broader impact on patients’ daily lives and psychosocial well-being remains incompletely understood.

**Objective:**

To identify psychosocial and other emerging dimensions of contemporary diabetes technology use among adults with T1D.

**Design, Setting, and Participants:**

This qualitative study used thematic analysis of free-text survey responses collected between August 1, 2024, and February 28, 2025, from adults (aged ≥18 years) with T1D receiving care within a single, large, academic health system in the US. Participants represented a range of ages, diabetes duration, and age at onset; most were users of CGMs and AID systems.

**Results:**

Of 5650 individuals who were invited to participate in the study, 945 eligible patients responded to the survey and 535 (mean [range] age, 48 [18 to ≥70] years; 282 [52.8%] female) provided free-text responses regarding the impact of diabetes technology on daily life that were included in the qualitative analysis. The median (range) diabetes duration was 25 (0 to ≥31) years. Overall, 508 (95.0%) were using CGMs and 369 (69.0%) were using insulin pumps. Participants described diverse experiences with modern diabetes technology, reflecting both benefits and burdens. In addition to established psychosocial themes, several novel concerns were identified: environmental impact from device-related waste, travel-related stress due to supply logistics and inconsistent airport security procedures, distress related to premature device failures and challenges navigating manufacturer and insurance replacement processes, and apprehension about digital security. These structural and societal factors were described as meaningful contributors to overall technology experience.

**Conclusions and Relevance:**

In this study of adults with T1D, contemporary diabetes technology affected more than glycemic control; environmental sustainability, administrative burden, travel logistics, and digital trust represented emerging dimensions of technology-dependent care. Addressing these concerns through patient-centered design, streamlined system processes, and transparent cybersecurity protections may enhance the long-term sustainability and acceptability of advanced diabetes technologies.

## Key Points

**Question:**

What psychosocial and emerging challenges are associated with contemporary diabetes technology use among adults with type 1 diabetes?

**Findings:**

In this qualitative study of 535 adults receiving care within a large academic health system, participants described several novel concerns underrepresented in the literature, including environmental impact of device-related waste, travel burden, distress surrounding device replacement and insurance navigation, and apprehension about digital security.

**Meaning:**

This study’s results suggest that modern diabetes technology introduces structural and societal challenges that warrant attention to improve the patient-centeredness and long-term acceptability of technology-dependent diabetes care.

## Teaser

This qualitative study of adults with type 1 diabetes examines their perspectives on how diabetes technology affects patients’ daily lives and psychosocial well-being.

## Introduction

Use of continuous glucose monitors (CGMs) and insulin pumps in type 1 diabetes (T1D) care has increased substantially during the past decade.^1,2^ CGMs provide real-time glucose monitoring, and contemporary insulin pumps, many of which are automated insulin delivery (AID) systems, adjust insulin administration in response to CGM data to better regulate glucose levels. Although the clinical benefits of these devices on glycemic control are well established,^3,4,5^ far less is known about their impact on patients’ daily lives.

Existing research often assesses these impacts using standardized measures (eg, Diabetes Distress Scale, Problem Areas in Diabetes, Diabetes Treatment Satisfaction Questionnaires, Hypoglycemia Fear Survey, and various quality-of-life instruments). Although many studies^6,7^ report improvements in diabetes distress, fear of hypoglycemia, and diabetes-specific quality of life, others^8,9^ highlight increased psychosocial burden, including alarm fatigue, cognitive burden, financial stress, and daily disruptions. Qualitative methods are well suited to capture the emotional, cognitive, and social dimensions of diabetes technology use that are not well captured by standardized scales. However, prior studies have been limited by focus on pediatrics, single device types, or evaluation of earlier-generation systems that do not reflect widespread AID adoption.^10,11,12,13,14,15,16,17,18,19,20^ As diabetes care becomes increasingly more technology dependent, understanding how these devices shape patients’ emotional well-being, daily functioning, and interactions with health systems is essential. Therefore, we conducted a large qualitative study of adults with T1D to identify emerging and potentially underrecognized psychosocial challenges of contemporary diabetes technology. Our aim was to evaluate patients’ perspectives on the impact of diabetes technology on their daily lives and psychological well-being.

## Methods

### Study Design

On review and approval of the study by the UCLA institutional review board, adults (aged ≥18 years) with T1D receiving care within the UCLA Health system were sent a single invitation via the patient portal between August 1, 2024, and February 28, 2025, to complete a survey. The survey assessed diabetes technology use, daily functioning, quality of life, and emotional well-being. Electronic informed consent was obtained from all participants before survey initiation. Participants were asked an open-ended question: “Tell the research team anything else you would like us to know about how your diabetes regimen or technology impacts your life.” Free-text responses to this question were analyzed qualitatively. This study is reported in accordance with the Standards for Reporting Qualitative Research (SRQR) reporting guideline.

### Qualitative Analysis

Free-text responses were exported from Qualtrics and imported into Dedoose, version 9.0.107 (SocioCultural Research Consultants LLC). A thematic approach was used, starting with open coding to identify patterns in the participant’s responses and followed by development of a codebook to categorize responses into themes. To ensure credibility and rigor, 2 researchers (E.S. and B.E.B.) independently coded each response and resolved any discrepancies through discussion. During the coding process, the researchers identified additional nuanced subthemes that extended beyond the initial codebook. As a result, the team incorporated these subthemes and conducted a second-pass recoding of all responses. This iterative approach ensured that even the most nuanced and complex perspectives regarding diabetes technology were accurately represented in the final analysis. Participant race and Hispanic ethnicity were derived from 2 survey items and classified based on combined responses (eg, White, non-Hispanic). If ethnicity was missing, participants were considered non-Hispanic. The other race category included American Indian or Alaskan native, multiracial participants, and those who selected “prefer not to say.” Race and ethnicity data were collected because they are important social constructs associated with differences in health care access, health care use, and health outcomes. These variables were included to characterize the study population, assess the generalizability of findings, and evaluate potential disparities in diabetes technology use and related outcomes across demographic groups.

## Results

Of the 5650 invited participants, 945 eligible participants responded, and 535 (mean [range] age, 48 [18 to ≥70] years; 282 [52.8%] female) provided qualitative responses included in this analysis (443 [82.8%] were technology related). The mean (SD) response length was 63 (65) words. Demographic characteristics and technology use of responders are presented in Table 1. There were minimal differences between those who responded to the free-text question and those who did not. Our qualitative analysis revealed 4 major themes: (1) psychosocial and emotional impact (n = 321), (2) daily functioning and lifestyle (n = 184), (3) environmental impact (n = 12), (4) device performance and user experience (n = 186), with some responses contributing to multiple themes. These categories encompassed several subthemes, which are elaborated on below (additional quotations can be found in Table 2).

**Table 1.** Characteristics of the Study Participants

Characteristic	No. (%) of participants (N = 535)
Gender	
Male	243 (45.5)
Female	282 (52.8)
Transgender or nonbinary	5 (0.9)
Prefer not to say or missing	5 (0.9)
Age group, y	
18-29	78 (14.6)
30-39	121 (22.7)
40-49	83 (15.5)
50-59	92 (17.2)
60-69	95 (17.8)
≥70	66 (12.4)
Race and ethnicity	
Asian or Pacific Islander, non-Hispanic	21 (3.9)
Black, non-Hispanic	12 (2.2)
Hispanic	59 (11.0)
White, non-Hispanic	347 (64.9)
Other^a^	96 (17.9)
Educational level	
High school diploma, GED, or less	15 (2.7)
Some college (no degree) or associate’s degree	108 (20.2)
Bachelor’s degree	196 (36.6)
Master’s degree	105 (19.6)
PhD, MD, or other advanced professional degree	41 (7.7)
Prefer not to say or missing	70 (13.1)
Diabetes duration, y	
0-5	48 (9.0)
6-10	59 (11.0)
11-15	58 (10.8)
16-20	66 (12.3)
21-25	63 (11.8)
26-30	53 (9.9)
≥31	188 (35.1)
CGM history	
Current use	508 (95.0)
Previously used	16 (3.0)
Never used	11 (2.0)
CGM type	
Dexcom G6	172 (33.9)
Dexcom G7	256 (50.4)
FreeStyle Libre 2	8 (1.6)
FreeStyle Libre 3	21 (4.1)
Eversence	0
Medtronic Guardian Connect	1 (0.2)
Medtronic Guardian Sensor 3	7 (1.4)
Medtronic Guardian Sensor 4	39 (7.7)
Other	4 (0.8)
Duration of CGM use, y	
0-5	195 (38.4)
6-10	220 (43.3)
≥11	93 (18.3)
Pump history	
Currently using	369 (69.0)
Previously used	41 (7.7)
Never used	125 (23.4)
Insulin pump type	
Tandem t:slim X2	204 (55.3)
Tandem Mobi	8 (2.2)
Omnipod Dash	13 (3.5)
Omnipod 5	81 (22.0)
Medtronic 630G	1 (0.3)
Medtronic 670G	4 (1.1)
Medtronic 770G	7 (1.9)
Medtronic 780G	43 (11.7)
iLet Bionic Pancreas	6 (1.6)
Other	2 (0.5)
Duration of pump use, y	
0-5	90 (16.8)
6-10	70 (13.1)
11-15	61 (11.4)
16-20	60 (11.2)
≥21	254 (41.5)

Abbreviations: CGM, continuous glucose monitor; GED, General Educational Development.

^a^ Other includes American Indian or Native Alaskan, multiracial, or prefer not to say.

**Table 2.** Additional Study Participant Quotations

Subtheme and valence	Quotation
**Psychosocial and emotional impact**	
General anxiety	
Positive	“Before I had access to the CGM, I almost died several times during my sleep due to hypoglycemia...After those very close calls, I became so anxiety ridden that I would miss a life-threatening low glucose episode in my sleep that I would set alarms every few hours during the night to check glucose with my glucometer. I never slept for more than 2 hours at once. I was always exhausted! With the CGM…I can finally sleep through the night without fear of my body silently failing me while I’m trying to rest. The improved sleep quality, lack of anxiety, [and] being able to rely on the CGM alarms rather than wake up every 2 hours, has been absolutely life changing.” (Female, 40s, 32 y with T1D)
Negative	“Continuously needing to make decisions, worry about changing sites, things falling off, ripping off, following up with insurance, continuously needing new supplies, is so tiresome and stops me from doing out priorities, I worry about going out alone, especially on adventurous things, walks, hikes, camping (can’t be alone) and constantly carrying back up supplies is burdensome.” (Female, 30s, <10 y with T1D)
Financial stress	
Negative	“I have some of the best insurance and still pay a lot of money for my supplies. This is also discouraging. I have taken up a second job working nights just to pay be able to pay for the new medical bills I received. My quality of life is sad, but I am healthy.” (Female, 30s, <10 y with T1D)
Visibility to others	
Positive	“I take pride in wearing my diabetes tech in public. I get some looks and questions at times, but the highlight is meeting another diabetic, especially when it is a child. I feel that being type 1 for almost 36 years, I have something to offer to the newly diagnosed or worn-out parents.” (Female, 40s, approximately 30 y with T1D)
Negative	“I’d like the CGM to be less visible. I need to wear the CGM on my arm and often with a large obvious colorful patch. It screams diabetes even if I don’t want to announce it to all.” (Female, 50s, <10 y with T1D)
Physical markers of long-term device wear	
Negative	“I was never told that fat accumulates around the pump insertion sites very quickly. I switch sites each time but my belly fat is increasing and I don’t know how to get rid of it.” (Female, 60s, approximately 30 y with T1D)
**Daily functioning and lifestyle**	
Sleep	
Positive	“[My pump and CGM] has helped reduce the mental fatigue of treatment decisions....I can sleep any night without the fear of having an undetectable low or high blood glucose. I no longer fear a low because I get alerts from my pump. (Female, 40s, approximately 40 y with T1D)
Negative	“The biggest pain point I have with my cgm is the frequency of notifications. For example, if I am asleep and have a low, the alarm will sound. Well then if I correct it the low, the alarm will keep sounding every 5 minutes until it goes up. At times after a correction, it can take 30 minutes to show a correction. At that point I’m awake.” (Male, 20s, >20 y with T1D)
Exercise	
Positive	“CGM has been a life changer. It gave me the confidence to get back to long runs. I ran a marathon prior to T1D, and I thought I’d never run one again when using a meter. CGM restored that opportunity.” (Male, 28, <10 y with T1D)
Negative	“Swimming can be a bit challenging since my current pump is not waterproof. In order to swim I must remove my pump, make sure it’s in a safe place and keep on eye on it since replacement cost would be in the thousands of dollars.” (Female, 40s, approximately 40 y with T1D)
Work and life	
Negative	“I wish there were more developments in technology at least the recognition of the issues alarms can pose in a work environment and in public. Of course, a job cannot retaliate or anything, but I work in entertainment, and occlusion alarms or low battery alerts from my pump have ruined takes before. I wish there was a way to acknowledge that I am manually monitoring and briefly turn the alarms off including emergency ones.” (Female, 20s, approximately 10 y with T1D)
Travel	
Negative	“Airport security with a pump is a major hassle. The pump companies worry about scanning machines damaging electronics, so the manual requires users to request a hand check. This slows everything down and confuses many screeners.” (Male, 30s, approximately 10 y with T1D)
**Environmental impact**	
Waste	
Negative	“Both [my CGM and pump] produce large amount of trash for every use.” (Male, 60s, approximately 20 y with T1D)
**Device performance and user experience**	
Impact of device inaccuracy	
Negative	“The alarms on the CGMs are horrible and significantly impacts my control. It will alarm over and over and over for no reason. It says my number is going low or high, but it isn’t, and I’ve already corrected for it but because it keeps alarming it makes it seem like I need to correct more. This sends me on a rollercoaster of corrections.” (Female, 30s, approximately 30 y with T1D)
Failures and connectivity	
Negative	“The [CGM1] is relatively new to me and not only did the very first sensor fail, but I’ve had more failures with it in the brief time I’ve been using it compared to the [CGM2], which seemed to have a much lower frequency of failures. This has increased my distress that my CGM will fail on me, particularly when I’m traveling or on my last sensor before getting a refill of my prescription.” (Female, 30s, approximately 20 y with T1D)
Replacement	
Negative	“The biggest challenge is getting the tech support people at the companies to replace pumps, sensors, CGMs, etc when I know they need to be replaced.”(Female, 70s, approximately 30 y with T1D)
Wearability	
Negative	“I wish they were smaller and way less expensive. Sometimes certain clothes are tough to wear. And it’s very inconvenient when they pop off or are ripped off by bumping my arm against a doorway, taking off my shirt, or simply sitting down on an airplane. This is frustrating because it requires a new pump or [CGM], time and place to set up again, and it is wasted money when they are removed in the middle of a cycle.” (Male, 20s, <10 y with T1D)
Security	
Negative	“I also don’t like apps that...map out every single bloods sugar test.” (Male, 40s, approximately 30 y with T1D)

Abbreviations: CGM, continuous glucose monitor; T1D, type 1 diabetes.

### Theme 1: Psychosocial and Emotional Impact

Participants described multiple ways in which the use or absence of diabetes technology influenced their mental and emotional well-being. Their experiences reflected both meaningful benefits and notable challenges. Within this overarching theme, 6 subthemes emerged: anxiety, financial stress, visibility to others, and physical markers of long-term wear.

#### Anxiety

Participants reported that diabetes technology could alleviate or heighten their anxiety. For some, the devices reduced emotional distress by offering real-time information and a greater sense of security. However, some participants shared that using diabetes technology introduced new sources of anxiety, particularly related to the constant influx of glucose data. As one participant stated, “Sometimes always knowing what our [glucose] numbers are can be information overload and can actually cause more distress than getting snapshots of our numbers and testing when we felt symptoms of hypo/hyperglycemia and after meals like we did when we used glucometers. Sometimes seeing the number all the time makes me worry more about my numbers vs worrying less.” (Female, aged in 30s, approximately 20 years with T1D)

#### Financial Stress

Diabetes technology can be costly, even for individuals with comprehensive insurance coverage. Participants described the financial burden of device supplies as a significant source of stress. One participant stated the following:

Cost to purchase the pump supplies is the most crippling effect for all of this. I feel a great relief when I can use my CGM and pump systems, but affording it makes me practically unable to afford anything else. I’m in huge debt from buying supplies monthly and through health insurance the diabetes supplies are not regularly affordable at all. (Male, aged in 30s, approximately 30 years with T1D)

#### Visibility to Others

Many participants explained that their discomfort with diabetes technology stems from how visible the devices make their condition. Although some users said they do not mind when people ask sincere, thoughtful questions, many shared that being stared at or receiving unsolicited comments makes them feel exposed, embarrassed, or uncomfortable. One participant stated, “I’m not embarrassed by my diabetes technology or what I need to manage it, but I didn’t enjoy constantly being asked if I was wearing a pager” (Male, aged in 50s, approximately 20 years with T1D)

#### Physical Markers of Long-Term Device Wear

Some participants described stress related to long-term physical changes at infusion and sensor sites, including fat accumulation and scarring, which affected both comfort and body image. As one participant stated, “My [CGM] and [pump] definitely help me have more convenience and control of my diabetes but the [pump] leaves behind dark scars every time so I’m no longer comfortable wearing anything any that exposes my thigh area or stomach as there are a large amount of scars from every site.” (Female, aged in 30s, approximately 25 years with T1D)

### Theme 2: Daily Functioning and Lifestyle

Participants described mixed experiences on diabetes technology and their daily lives. Some described technology as an invaluable asset, whereas others found it intrusive and disruptive. Subthemes that emerged include sleep, exercise, work or school, and travel.

#### Sleep

Many participants shared that diabetes technology can be particularly disruptive at night, with alarms leading to frequent awakenings. One participant stated, “I wake up 2-4 times a night from my CGM alerts, often with high blood sugars keeping me up or forcing me to go for a walk, followed by a low later on that I have to wake up from and have sugar. And then I’m supposed to wake up and go to work like everyone else – it’s a stressful life.” (Male, aged in 20s, approximately 25 years with T1D)

#### Exercise

Some participants who use diabetes technology and engage in physical activity noted that it made it more practical to exercise, particularly with the ability to monitor glucose in real time. Others noted challenges with keeping their devices on body. One participant said, “Pumps are very heavy and fall off when I sweat, which means I cannot exercise as much or as rigorously as I need to in order to be as healthy as I want to be. Simply gardening or walking/hiking on a hot day causes the pump to fall off. I cannot count on swimming as a regular means of exercise, as the pump and CGM cannot withstand that kind of water exposure.” (Female, 50s, approximately 40 years with T1D)

#### Work and School

Participants described challenges managing diabetes technology in professional settings, where alarms and alerts could be disruptive or impractical. In some cases, the inability to silence or modify alerts interfered with job performance, leading individuals to miss alarms or adjust device use to accommodate workplace demands. One participant said, “I work on a sound stage, so I 100% can’t have an app or pump that I can’t silence. [My] pump has a vibration function which works for me, but because my phone vibrates…I don’t always catch high or low blood sugars in time due to me missing the alarms...” (Male, aged in 40s, approximately 40 years with T1D)

#### Travel

Travel was a notable challenge, with participants describing the need to maintain extra supplies, challenges navigating inconsistent Transportation Security Administration screening procedures, and difficulty managing uncertainties about device safety during airport screening. Participants described these travel issues as follows:

When traveling it causes some stress as [I] have to remember to take extra supplies in case sensor or insertion sets fail early. When going through airport security, I get mixed messages from manufacturers and airport security which security machines as safe to go through. I have to be vigil[ant] on making sure I have enough supplies on hand and make sure I get my CGM and pump supplies ordered timely. (Male, aged in 60s, <10 years with T1D)

Airport security can be a real challenge. [My] airport is terrible in recognizing the equipment and their response to a request for an opt out pat down is rude and harassing. (Female, aged in 60s, approximately 40 years with T1D)

I still struggle when I travel, having to carry all the extra supplies with me and worrying about the airlines giving me a hard time about it. It is the most stressful aspect of traveling. (Female, aged in 50s, approximately 25 years with T1D)

### Theme 3: Environmental Impact

An unexpected theme was concern about the environmental impact of diabetes technology. Participants expressed guilt over the volume of nonrecyclable waste generated by routine device use and wished for more reusable or recyclable options as follow:

I do wish there was a way to recycle the sensor applicator; I don’t feel great about the amount of medical waste I am producing by using a CGM. (Female, aged in 50s, <10 years with T1D)

Worry over nonrecyclable waste of device parts and packaging. (Female, aged in 30s, approximately 10 years with T1D)

I also have a strong dislike for the amount of waste these devices create and lack of proper instruction for disposal. (Nonbinary, aged in 50s, <10 years with T1D)

### Theme 4: Device Performance and User Experience

Device performance and user experience strongly influenced satisfaction. Although many valued the convenience and improved management, others reported challenges with device accuracy, difficulties obtaining replacements for early failures, wearability concerns, and fears about data security.

#### Impact of Device Inaccuracy

Despite overall benefits, many participants reported concerns about CGM inaccuracies. Unexpected high readings or false low alerts complicated treatment decisions and undermined trust in the technology. One participant stated,

“I don’t always feel confident that my CGM is accurate. For example, I was getting ‘urgently low’ alerts but didn’t feel like I do when I am low. I checked my sugar with finger prick and my sugar was 400. If I would have corrected for the ‘urgent low’ I could have easily gone into DKA [diabetic ketoacidosis]. I do tend to go based off my symptoms and gut feeling then feeling totally confident and ‘worry free’ with using the CGM.” (Female, 30s, approximately 20 years with T1D)

#### Failures and Connectivity

Participants described fear and frustration when CGMs or pumps malfunctioned. Site failures, sensor errors, and connectivity disruptions left users without reliable glucose data or clarity about insulin delivery and at times led to hyperglycemic emergencies. One participant said, “My pump [site] failed once, and I was in a complete panic. I didn’t have a backup with me (lesson learned), and by the time I got home and changed my [site], I was in ketoacidosis and had to go to the hospital. I worry about that kind of thing frequently.” (Female, aged in 60s, approximately 40 years with T1D)

#### Replacements

When device components failed, participants often had to contact manufacturers for replacements. Many described this process as time-consuming and stressful, especially because insurance restrictions prevented them from keeping backup supplies on hand. Participants expressed frustration with long customer service interactions, delays in receiving replacements, and reliance on insurance and manufacturer’s discretion, which left them feeling vulnerable and constrained, as follows:

I spend hours on the phone with [the manufacturers] when a piece of equipment goes wrong or needs to be replaced because insurance won’t allow me to have any backups on hand; therefore, I have to call one of those companies to prove I need an emergency replacement. (Male, aged in 30s, approximately 20 years with T1D)

I also get a lot of lost sensor errors which very much stresses me out – or the sensor doesn’t last the full 10 days, and I have to call for replacements which is deeply time-consuming. (Female, aged in 30s, approximately 25 years with T1D)

With the occasional CGM or pump failure, [dealing with insurance, prescriptions, or orders] makes it extremely hard to live with diabetes because you are sometimes at a mercy of [an] organization, which does not care and views you as a number, and does not have adequate ways of getting you supplies you need, ie, delays with authorizations, [CGM manufacturers] only replacing 3 failed systems for free per year (while you are not in control of the quality), insurance not covering a replacement or extra supplies for backup so you are living order to order. (Female, aged in 30s, <10 years with T1D)

#### Wearability

Many users reported wearability challenges, particularly with tubed pumps, describing bulkiness, difficulty concealing devices, and pumps catching on clothing. Women noted added difficulty wearing dresses or pocketless outfits. Despite valuing the technology, participants desired slimmer, more adaptable designs. As one participant described, “I feel the biggest impact is not the technology, or the settings, but the design of the pumps and CGMs. It’s like clothing, but it’s a one size fits all in how one wears it. Yes, wearing it, is a hassle on my end having a tube and heavy pager like box hanging of the pants, in my pocket, on my shirt.” (Male, 50s, approximately 30 years with T1D)

#### Security

Some participants expressed reluctance to use devices that rely on smartphone integration, citing concerns about data privacy, potential misuse of personal health information, and vulnerability to hacking. These concerns led some to prefer standalone devices or consider discontinuing certain technologies altogether. Participants described these security issues as follows:

Please convince [CGM company] (a brilliant and decent company) to issue their newer CGMS with an independent monitoring device. I will never use one that requires my phone, and the sharing of my very personal data. (Female, aged in 60s, approximately 25 years with T1D)

I do not like for my pump or CGM to be paired with apps. I am concerned about hacking and prefer a dedicated medical device. I am not interested in any medical technology that relies solely on my phone for it to operate. (Female, aged in 40s, approximately 40 years with T1D)

I will probably never get a CGM that uses your cellphone (sells your health information), so will probably have to go back to fingerstick when my current CGM breaks. (Female, aged in 60s, approximately 25 years with T1D)

## Discussion

Our study identified diverse experiences among adults with T1D using modern diabetes technology. Our findings were consistent with prior literature^10,11,12,13,14,15,16,17,18,19,20^ documenting mixed psychosocial effects, with many patients reporting both positive and negative impacts on their quality of life and lifestyle. The current study highlights 4 less-explored contemporary themes: environmental concerns related to device waste, travel burden, distress surrounding replacement navigation, and digital security fears.

The extent to which participants expressed concern about the environmental consequences of diabetes technology has not been included in prior reports and is a topic of great national and international interest.^21,22,23,24,25,26^ Participants described concern regarding the substantial volume of nonrecyclable plastic waste generated by routine device use. As CGM and AID adoption continues to expand globally,^1,2^ the cumulative environmental footprint will likely increase. These findings suggest that sustainability represents an emerging ethical and societal dimension of diabetes care that warrants consideration by device manufacturers and policymakers.

Travel emerged as another underexamined source of stress. Participants described travel-related stress, including maintaining backup supplies and navigating inconsistent airport security procedures. Although clinical recommendations for travel preparation exist,^27^ travel as a psychosocial dimension of modern diabetes technology use is not widely recognized. As diabetes management becomes increasingly device dependent, standardized travel guidance, advocacy for standard Transportation Security Administration screening procedures, and improved device compatibility within screening methods may help mitigate this burden.

Distress related to obtaining replacement devices and supplies after premature failures was another salient and underreported theme. Prolonged interactions with manufacturers and insurance restrictions on maintaining backup supplies compounded the stress associated with device malfunction. Although noted in preliminary reports,^28,29^ this topic remains minimally represented in the peer-reviewed literature and reflects structural and administrative barriers that shape patients’ experience with technology use.

Some participants expressed concern about cybersecurity and reliance on smartphone integration. As diabetes technologies increasingly rely on wireless connectivity, cloud-based data sharing, and app-integrated ecosystems, concerns regarding privacy and digital security may influence both adoption and sustained use. Despite rapid technological advancement, few studies have examined patient perceptions of cybersecurity in diabetes care. Transparent communication from manufacturers regarding cybersecurity safeguards, along with robust regulatory oversight, may be essential to maintaining trust in connected medical devices.

### Strengths and Limitations

This study has several strengths. This study, to our knowledge, represents one of the largest qualitative analyses of psychosocial experiences with contemporary diabetes technology, allowing us to reflect a broad range of responses and experiences, including less common or more nuanced perspectives that may not be captured in smaller samples. The sample included participants with diverse ages, diabetes duration, and age at onset, including both childhood and adult-onset T1D, and was not limited to a single device type, enhancing relevance to current diabetes care.

This study also has limitations. Responses were based on an open-ended survey question rather than interviews, which may enhance candor^21^ but limits opportunities for probing and clarification. Voluntary participation may also introduce response bias. Additionally, this sample was from a single academic health system and was not fully representative of the broader US T1D population, with most participants identifying as White and reporting high educational attainment. The sample was also skewed toward experienced technology users and specific device brands. These characteristics may limit the generalizability of findings to more diverse populations, including individuals from underserved backgrounds, those with lower educational attainment, and those with less experience with diabetes technologies. As such, the psychosocial experiences observed in this study may not fully capture the complexity encountered in more heterogeneous practice settings.

## Conclusions

This study of adults with T1D who largely use diabetes technology suggests that the impact of contemporary diabetes technology extends beyond glycemic outcomes and constructs captured by traditional psychosocial scales. Environmental sustainability, travel logistics, administrative burden, and digital trust represent emerging dimensions of modern diabetes care. Addressing these concerns will require coordinated efforts among clinicians, manufacturers, insurers, and policymakers to ensure that continued technological innovation aligns with patient needs and practical experiences.

## Notes

Supplement.
Data Sharing Statement
