# Supplementary material for: Contemporary Diabetes Technology and the Patient Experience
Source: JAMA Netw Open. 2026 Jul 21;9(7):e2624260. doi: 10.1001/jamanetworkopen.2026.24260 (PMC13389783; doi:10.1001/jamanetworkopen.2026.24260)
Supplement: Supplement. — Data Sharing Statement [file jamanetwopen-e2624260-s001.pdf]

Data Sharing Statement

Everett. Contemporary Diabetes Technology and the Patient Experience. *JAMA Netw Open*. Published July 21, 2026. doi:10.1001/jamanetworkopen.2026.24260

Data
Data available: No
